# Resident wellness in dermatology postgraduate medical education in Kuwait: A cross-sectional study

**DOI:** 10.1016/j.jdin.2025.07.001

**Published:** 2025-07-15

**Authors:** Hasan Ashkanani, Fatima AlJuaidi, Fahad AlQenae, Abeer AlBazali

**Affiliations:** a Assistant Registrar, Department of Dermatology, Al-Amiri Hospital, Kuwait City, Kuwait; b Senior Specialist, Department of Dermatology, Al-Adan Hospital, Kuwait City, Kuwait; c Registrar, Department of Dermatology, Al-Adan Hospital, Kuwait City, Kuwait; d Consultant, Department of Dermatology, Al-Farwaniyah Hospital, Kuwait City, Kuwait

**Keywords:** anxiety, burnout, dermatology, distress, education, epidemiology, leadership, residency, stress, wellness

*To the Editor:* Physician burnout is associated with increased medical error rates and compromised patient care.^1^^,^^2^ Dermatology residents, akin to trainees in other medical specialties, remain vulnerable to the demands of postgraduate training, with approximately one-third meeting established criteria for burnout.^3^ This study presents the first national assessment of dermatology resident wellness in Kuwait, evaluating access to support resources, reported stressors, and prevalence of psychological distress among trainees.

In late 2023, an anonymous, 16-item validated survey adapted from a published study^4^ was administered to all 42 residents enrolled in the Kuwait Board of Dermatology program. The survey assessed domains including availability of wellness infrastructure, departmental culture, and self- or peer-reported experiences of burnout, depression, or suicidal ideation. The response rate was 100%.

Only 2 respondents (4.8%) reported access to free, confidential mental health counseling, and 92.9% indicated that any such counseling should remain strictly confidential from program leadership. A majority (78.6%) perceived little to no emphasis on wellness within the training environment, and only 7.1% reported the presence of a formal wellness initiative. Key reported stressors included excessive academic workload (71.4%), lack of formal wellness programming (61.9%), limited access to counseling services (59.5%), and administrative burden (59.5%). Dermatology-specific challenges were also prominent: 85.7% identified board examination preparation as a major stressor. Notably, 45.2% reported personal or peer experiences with serious psychological distress, and 38.1% had witnessed clinical care compromised due to resident fatigue or burnout. Senior trainees reported significantly higher levels of stress related to workload and performance expectations compared to their junior counterparts (*P* < .05).

Dermatology trainees in Kuwait face substantial wellness challenges, with limited institutional support and a notable burden of psychological distress. These findings are consistent with international literature reporting burnout rates of 30% to 50% among residents,^2^^,^^4^ underscoring the global relevance of this issue. Surveyed residents prioritized systemic interventions over isolated measures, with one-third advocating for structured stress management education and one-third recommending the establishment of a formal resident wellness committee. We strongly encourage residency leadership to implement structured wellness curricula, enhance access to confidential mental health resources, and foster a culture that proactively addresses burnout. Such efforts are essential not only for preserving trainee well-being but also for upholding standards of patient safety and clinical excellence.^5^

## Conflicts of interest

None disclosed.
